# Proton therapy of a pregnant patient with nasopharyngeal carcinoma

**DOI:** 10.1016/j.ctro.2022.04.014

**Published:** 2022-05-04

**Authors:** Joosje H. Heimovaara, Jeroen Blommaert, Jeffrey Free, René A. Bolt, Elske M. Gort, Tom Depuydt, Cristina Boso Martinez, Mirthe H. Schoots, Mathilde van Gerwen, Marry van den Heuvel-Eibrink, Johannes A. Langendijk, Carolien P. Schröder, Frédéric Amant, Sanne J. Gordijn, Edwin Oldehinkel

**Affiliations:** a Department of Oncology, KU Leuven, Leuven, Belgium; b Department of Gynecologic Oncology, Netherlands Cancer Institute and Amsterdam University Medical Center, Amsterdam, the Netherlands; c Department of Radiation Oncology, University Medical Center Groningen, University of Groningen, Groningen, the Netherlands; d Department of Radiation Oncology, University Hospitals Leuven, Leuven, Belgium; e Department of Pathology and Medical Biology, Pathology Section, University Medical Center Groningen, University of Groningen, Groningen, the Netherlands; f Princess Máxima Center for Pediatric Oncology, Utrecht, the Netherlands; g Department of Medical Oncology, University Medical Centre Groningen, University of Groningen, Groningen, the Netherlands; h Department of Medical Oncology, Netherlands Cancer Institute and Amsterdam University Medical Center, Amsterdam, the Netherlands; i Department of Gynecologic Oncology, University Hospitals Leuven, Leuven, Belgium; j Department of Obstetrics and Gynecology, University Medical Centre Groningen, University of Groningen, Groningen, the Netherlands

## Abstract

**Background and purpose:**

Radiotherapy during pregnancy is rarely administered due to lack of data and practical challenges. This is the first detailed report of proton therapy as cancer treatment for a pregnant patient with nasopharyngeal carcinoma.

**Materials and methods:**

Pencil beam scanning proton therapy was prescribed to a pregnant patient to a total dose of 70 Gy (RBE) to the therapeutic CTV and 54.25 Gy to the prophylactic CTV, delivered in 35 fractions with a simultaneous integrated boost technique.

**Results:**

Phantom measurements showed a thirty-fold decrease in fetal radiation dose when using proton compared to photon therapy, with a total fetal dose of 5.5 mSv for the complete proton treatment, compared to 185 and 298 mSv for the photon treatment with and without lead shielding, respectively. After adminstering proton therapy during pregnancy, at 39 weeks of gestation, a healthy boy with a birthweight on the 83th percentile was delivered. Pediatric follow-up at 2 months of age of the offspring showed normal growth and age-adequate motor development with no signs of neurological problems. MR follow-up of the tumor 3 months after the end of treatment showed complete remission.

**Conclusion:**

This case demonstrates the potential of proton therapy for treatment during pregnancy.

Compared to photon therapy, proton therapy can significantly limit fetal dose, while simultaneously offering a more optimized treatment to the patient.

## Highlights

• First in human report of IMPT in a pregnant patient with nasopharyngeal carcinoma.
• Physical phantom measurements showed a thirty-fold decrease in fetal radiation dose when using IMPT compared to photon therapy.
• IMPT during pregnancy did not result in obstetrical or neonatal complications.

## Introduction

Cancer complicates about 1 in 1000 to 2000 pregnancies [1]. With growing evidence regarding the safety of cancer treatment during pregnancy, approximately 69% of pregnant patients are currently treated with surgery or cytotoxic modalities [2]. However, only 1–3% of pregnant cancer patients receive radiotherapeutic treatment [2], [3]. Although guidelines indicate that this is safe when the fetal dose is kept below 100 mSv, radiation oncologists are hesitant to treat during pregnancy due to practical challenges and lack of data [2], [3], [4], [5].

Pencil beam scanning intensity modulated proton therapy (IMPT) has recently provided new opportunities for radiotherapy during pregnancy. So far, three pioneering case studies have reported on the feasibility of IMPT during pregnancy [6], [7], [8]. However, these studies did not compare the fetal radiation dose with state-of-the-art photon therapy and scarcely discussed the oncological and obstetrical outcomes. One Monte Carlo simulation study comparing simulated (phantom) fetal dose during photon therapy with IMPT for a brain tumor showed a ten-fold reduction in fetal radiation dose [9]. This study was performed using an artificial tumor and patient model and was not validated by physical measurements.

In this study, we describe the outcomes of a pregnant woman receiving IMPT for a nasopharyngeal carcinoma and the outcomes of her child, and calculated the fetal radiation doses during proton and photon therapy using phantom measurements.

## Case

A 33-year-old Asian pregnant woman, gravida 4, para 1, 24 weeks of gestation with no relevant medical history and normal glucose tolerance test, presented with a two-month history of frequent epistaxis, nasal obstruction and palpable mass in the neck. Naso-endoscopy showed an exophytic mass located in the right nasopharynx and biopsy confirmed non-keratinizing squamous cell carcinoma, Epstein-Barr virus (EBV) positive. Head and neck magnetic resonance imaging (MRI) showed a lesion in the right nasopharynx (approximately 26 × 32 x 30 mm), extending over the midline to the left side. There were bilateral retropharyngeal pathological lymph nodes as well as in the right neck, level II, III and V, with a maximal short axis diameter of 20 mm. Computer tomography (CT) of the thorax did not show distant metastases. Definitive staging was a cT1N1M0 (according to TNM 8th edition) EBV + nasopharyngeal carcinoma [10]. The patient was referred to an academic medical hospital with a proton facility and experience in the field of cancer and pregnancy, where radiotherapy with IMPT was indicated after multidisciplinary consultation.

The patient was immobilized using a 5-point thermoplastic mask and underwent a non-contrast enhanced planning CT (pCT) and MRI. Gross tumor volume contained the primary tumor and pathological lymph nodes. Clinical target volume (CTV) was obtained by adding a 5-mm margin in all directions adjusted to anatomical structures for the therapeutic CTV and a 10-mm margin for the prophylactic (elective) CTV. The prophylactic CTV also included lymph node levels II, III, IVa, Vab and VIIa according to consensus guidelines [11]. Prescribed radiation dose was 70 Gy (RBE) delivered in 35 fractions of 2 Gy to the therapeutic CTV and 54.25 Gy (RBE) in 35 fractions of 1.55 Gy to the prophylactic CTV using a simultaneous integrated boost technique and CTV-based robust treatment planning.

**Fig. 1:**
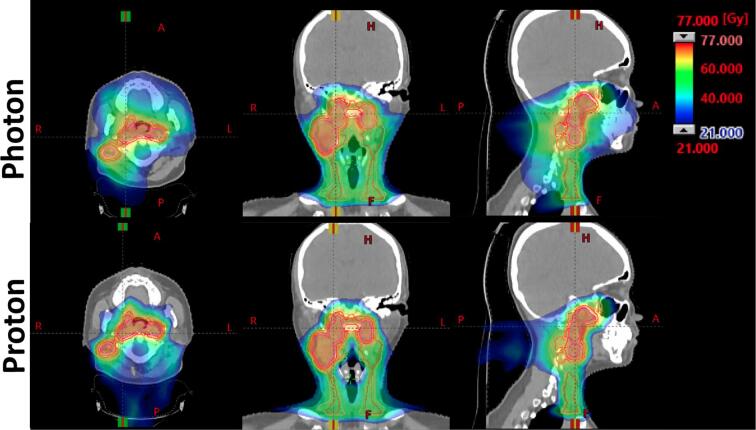
Proton and photon treatment plans. The top three panels show an axial, coronal and sagittal plan of the VMAT photon plan. The lower three panels show the same planes for the IMPT plan.

**Fig. 2:**
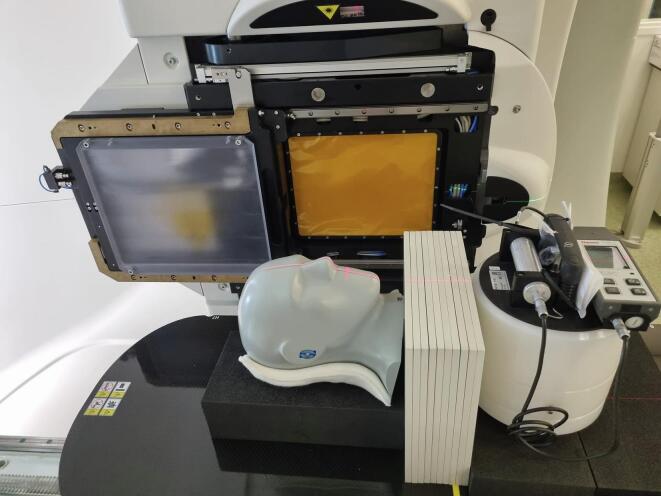
Phantom set-up for fetal dose estimation during proton treatment.

**Fig. 3:**
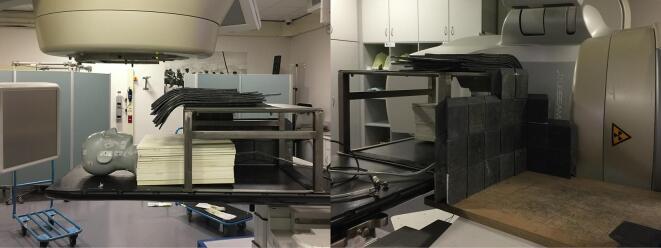
Phantom set-up for fetal dose estimation during the VMAT photon treatment.

Before starting IMPT, fetal radiation dose was compared between proton and photon therapy plans (Table 1, Fig. 1) using phantom measurements. The palpable distance between fetus and edge of the CTV before start of treatment was 30 cm. Accounting for dose uncertainties and fetal growth during treatment, fetal radiation dose was measured at 20 cm from the caudal border of the CTV. The same phantom set-up (Fig. 2, Fig. 3) was used for fetal radiation dose estimation in both proton and photon plans, consisting of a CIRS proton therapy head phantom (model 731-HN) and RW3 solid water plates for thorax and abdomen. For the proton plan. fetal dose was estimated using the FHT 762 Wendi*-*2 detector, placed with its center at 20 cm from the caudal border of the CTV using a vertical stack of 30 by 30 cm solid water plates. The Wendi-2 detector is designed to measure H*(10) for neutrons up to 5 GeV as well as gamma radiation. For the VMAT photon plan, TLD-100 chipstrate detectors were used to estimate the fetal radiation dose, placed in a 16 cm horizontal stack of 40 by 40 cm solid water plates at a depth of 7 cm and at 20 cm from the caudal border of the CTV. The TLD’s were calibrated in absorbed dose against an FC65-G Scanditronix-Wellhöfer Farmer-type ionisation chamber in a stack of solid water plates, using a 6MV beam and a 10 by 10 cm field at a depth of 10 cm and an SSD of 90 cm.

**Table 1:** Treatment parameters for the proton and photon plans.

	Proton	Photon
Treatment system	IBA Proteus Plus	Varian Truebeam STX
Technique	Pencil beam scanning	6 MV VMAT
TPS	Raystation v9A	Eclipse v16.1
Couch rotation	0°	10°
Gantry rotation	50, 85, 160, 200, 280 and 315°	90 to 280° back-and-forth
Range shifter (4cm WET)	50, 85, 280 and 315°	NA
Monitor units	1792.37 MU	373 MU
Lead shielding	None	7.7 cm frontal and 5 cm left
Detector for fetal dosimetry	FHT 762 Wendi-2	TLD-100 chipstrate
Estimated fetal dose	5.5 mSv	185 mSv

A Pencil Beam Scanning (PBS) IMPT plan was made using the Raystation 9A treatment planning system (RaySearch Laboratories, Sweden) for an IBA Proteus Plus (IBA, Belgium) delivery system. The plan setup was performed according to clinical routine due to the lack of specific guidelines for treatment of pregnant women. The proton plan consisted of 6 beams without couch rotations and gantry angles of 50, 85, 160, 200, 280 and 315° with a total of 1792.37 MU. Beams at gantry angle 50, 85, 280 and 315° were equipped with a range shifter of 4 cm water equivalent thickness (WET). The treatment plan was robustly optimized using the RayStation Monte Carlo dose engine with an uncertainty of 1.0%, an isotropic setup uncertainty of 3.0 mm and a range uncertainty of ± 3.0%. The final plan was evaluated on CTV coverage based on the voxel-wise minimum and voxel-wise maximum dose maps constructed out of the 28 scenarios (i.e. 14 isotropic setup scenarios with maximum setup uncertainty of 3.0 mm for two (±3.0%) range uncertainty scenarios) [12]. The 6 MV VMAT photon plan was created (Eclipse v16.1, Varian, USA) for a Varian Truebeam STX using a PTV with 3 mm margin around the CTV, according to clinical routine. The plan consisted of a back-and-forth arc of 90 and 290° at 10° table rotation, to avoid irradiating from angles where lead shielding was impossible. In line with AAPM guidelines, monitor units were limited to 373 MU and frontal 7,7cm and left 5 cm thick abdominal lead shielding was used [13]. Phantom measurements showed an estimated total fetal radiation dose of 5.5 mSv for the proton treatment, of which 4.6 mSv due to neutrons. For photon treatment the estimated total fetal doses were 185 and 298 mSv, respectively with and without lead shielding.

IMPT started at 27 weeks of gestation. The patient underwent daily orthogonal kilovoltage X-ray imaging and cone beam CT (CBCT) during treatment to ensure correct patient setup each day, attributing to an additional fetal dose of 0.08 mSv (2.25 μSv per fraction), as measured by the Wendi-2 detector. Additionally, the patient received weekly repeat CTs (rCT) to verify adequate dose coverage and dose to organs at risk (OARs). Plan adaptations were not required. The weekly repeat and planning CTs accounted for an additional fetal radiation dose of 0.21 mSv, with no abdominal shielding being used [14]. Accounting for both treatment and imaging, the total fetal radiation dose was 5.8 mSv.

The course of pregnancy from an obstetric perspective was uneventful, with fetal growth along the 70th percentile and normal umbilical artery Dopplers. During treatment, patient developed grade 2 mucositis and grade 3 dermatitis (according to the Common Terminology Criteria for Adverse Events version 4.0 (CTCAE 4.0) for which additional symptomatic pain relief was started at 29 weeks of gestation [15]. Lidocain spray 10% 3 times a day and additional tramadol 50 mg two times a day resulted in efficient pain relief. Six weeks after the last fraction, symptoms were completely resolved.

At 39 weeks of gestation, four months after diagnosis and eight weeks after end of treatment, the patient gave birth to a healthy boy after spontaneous delivery. The boy weighted 3820 g (83th percentile) and measured 53 cm in length and 36 cm in head circumference [16]. APGAR scores were 9 and 10 at respectively 1 and 5 min. No congenital or neurological abnormalities were observed in the child. The placental weight was 445 g (normal for gestational age) and general pathological examination showed no abnormalities. Microscopic placental examination according to the Amsterdam criteria showed more than 45 avascular villi, compatible with the diagnosis of a high-grade fetal vascular malperfusion (FVM) [17]. No maternal vascular malperfusion, chronic villitis or chronic intervillositis were found. The maturation of the parenchyma was slightly immature for the given gestational age.

Follow-up at two months of age showed normal general health, development and growth of the boy. No signs of neurological problems were observed. Long-term follow-up of this boy is planned to evaluate possible long-term side effects. Three months after treatment, the patient underwent an MRI of the head and neck which showed a complete remission. Six months after treatment no clinical signs of recurrent disease were observed.

## Discussion

Here we present the potential of IMPT for treatment during pregnancy and show a significantly reduced fetal radiation exposure during IMPT compared to photon therapy, while simultaneously offering a more conformal treatment to the patient.

The FVM found in the placenta is a common lesion related to fetal growth restriction and adverse perinatal outcomes, however the abnormalities of FVM are also described in uneventful pregnancies with normal outcome [18]. Fetal growth restriction did not occur in our case as the birthweight was at the 83rd percentile after intrauterine growth along the 70th percentile during pregnancy. FVM can be caused by damage to or compression of fetal vessels [17], [19], [20]. No direct explanation for the avascular fetal villi was found. Although it has been suggested that irradiation can cause maternal vascular malperfusion (MVM), no signs of MVM were observed. Whether irradiation causes direct damage to the villous vasculature is yet unclear [21]. In vitro and animal studies need to be conducted to investigate the plausible pathophysiological mechanisms of fetal radiation on FVM.

The fetal radiation dose during proton therapy was observed to be dominated by secondary neutrons, accounting for 84% of the dose, which cannot be properly shielded using lead. Moreover, as PBS does not use collimation to shape the beam, less head scatter can be expected and therefore less need for abdominal shielding. This in contrast to photon treatment, where we observed a 38% reduction in fetal dose when applying lead shielding. Next to that, we minimized the fetal dose during photon treatment by limiting the treatment angles and MU. These modifications of the photon treatment provide additional practical challenges for treatment planning and execution, which was not the case for proton treatment.

Currently, the implementation of proton therapy during pregnancy faces several hurdles. First, neutron dominated out-field radiation generates uncertainties on the placental and fetal radiation dose and necessitate the use of large detector volumes. This can be solved by using more advanced smaller-sized detectors, which in turn allow for more realistic phantom geometries. Alternative, validated computational models could offer adaptable and patient-specific computation of fetal doses. Second, there is a lack of data on placental development and function, as well as on long-term outcomes in children born after radiotherapy during pregnancy. Clinicians should therefore be encouraged to report known cases and register pregnant patients receiving proton therapy in national and international registries. Third, in contrast to photon therapy, guidelines on the use of proton therapy during pregnancy are still to be established [22]. In conclusion, future research should improve the accuracy of the fetal neutron dose estimation during proton therapy, leading to practical guidelines and standardization on the use of proton therapy during pregnancy.

## Funding

J. Blommaert is an aspirant researcher for the Research Foundation Flanders (FWO 11B9919N).

## Declaration of Competing Interest

The authors declare the following financial interests/personal relationships which may be considered as potential competing interests: Dr. Langendijk reports personal fees from IBA and RaySearch paid to the UMCG Research BV. The department of Radiation Oncology has Research Collaboration with IBA, RaySearch, Elekta, Siemens, Leoni and Vision RT. Alle these are outside the submitted work. All other authors report no conflicts of interest.
